# miR-125a-5p/miR-125b-5p contributes to pathological activation of angiotensin II-AT1R in mouse distal convoluted tubule cells by the suppression of Atrap

**DOI:** 10.1016/j.jbc.2023.105478

**Published:** 2023-11-21

**Authors:** Keigo Hirota, Akio Yamashita, Eriko Abe, Takahiro Yamaji, Kengo Azushima, Shohei Tanaka, Shinya Taguchi, Shunichiro Tsukamoto, Hiromichi Wakui, Kouichi Tamura

**Affiliations:** 1 Department of Medical Science and Cardiorenal Medicine, Yokohama City University Graduate School of Medicine, Yokohama, Japan; 2 Department of Investigative Medicine, Graduate School of Medicine, University of the Ryukyus, Okinawa, Japan

**Keywords:** miR-125a-5p/miR-125b-5p, angiotensin II, AT1R, ATRAP–Atrap, mouse distal convoluted tubular cells

## Abstract

The renin–angiotensin system plays a crucial role in the regulation of blood pressure. Activation of the angiotensin II (Ang II)–Ang II type 1 receptor (AT1R) signaling pathway contributes to the pathogenesis of hypertension and subsequent organ damage. AT1R-associated protein (ATRAP) has been identified as an endogenous inhibitory protein of the AT1R pathological activation. We have shown that mouse Atrap (Atrap) represses various Ang II–AT1R-mediated pathologies, including hypertension in mice. The expression of human ATRAP (ATRAP)/Atrap can be altered in various pathological states in humans and mice, such as Ang II stimulation and serum starvation. However, the regulatory mechanisms of ATRAP/Atrap are not yet fully elucidated. miRNAs are 21 to 23 nucleotides of small RNAs that post-transcriptionally repress gene expression. Single miRNA can act on hundreds of target mRNAs, and numerous miRNAs have been identified as the Ang II–AT1R signaling–associated disease phenotype modulator, but nothing is known about the regulation of ATRAP/Atrap. In the present study, we identified miR-125a-5p/miR-125b-5p as the evolutionarily conserved miRNAs that potentially act on ATRAP/Atrap mRNA. Further analysis revealed that miR-125a-5p/miR-125b-5p can directly repress both ATRAP and Atrap. In addition, the inhibition of miR-125a-5p/miR-125b-5p resulted in the suppression of the Ang II–AT1R signaling in mouse distal convoluted tubule cells. Taken together, miR-125a-5p/miR-125b-5p activates Ang II–AT1R signaling by the suppression of ATRAP/Atrap. Our results provide new insights into the potential approaches for achieving the organ-protective effects by the repression of the miR-125 family associated with the enhancement of ATRAP/Atrap expression.

Hypertension is one of the most common complications worldwide, predisposing health problems and affecting various organs. In 2010, 31.1% of the world's adult population (1.39 billion people) had hypertension (1). Among the pathways involved in the development of hypertension, the renin–angiotensin system (RAS) plays a crucial role; particularly, the angiotensin II (Ang II)–Ang II type 1 receptor (AT1R) signaling pathway directly affects the arterial constriction (2), tubular sodium reabsorption (3, 4, 5), the release of aldosterone, a mineralocorticoid (6, 7), and the induction of insulin resistance (8). Activation of the Ang II-AT1R signaling pathway at local sites contributes to the pathogenesis of hypertension, renal diseases associated with oxidative stress, and fibrotic conditions (9, 10). Consequently, RAS inhibitors are widely utilized as key drugs due to their antihypertensive and organ-protective effects (11). However, excessive inhibition of the RAS has been associated with adverse events including hypotension, hyperkalemia, and renal damage (12, 13).

AT1R-associated protein (ATRAP/Atrap) is identified as a direct binding protein of AT1R. ATRAP/Atrap acts as an endogenous inhibitory protein of the pathological AT1R hyperactivation at local tissue sites (14, 15, 16, 17, 18). For instance, Atrap overproduction (transgenic mice) or depletion (knockout mice) displays repressive or enhancement effects for Ang II–AT1R-mediated hypertension, cardiac hypertrophy, vascular injury, and insulin resistance by blocking pathological activation of AT1R without the alteration of baseline status. Hence, ATRAP/Atrap is a potential therapeutic target of pathological AT1R signaling activation without excessive inhibition.

Understanding how the ATRAP/Atrap expression is regulated is essential because of its protective function against harmful Ang II–AT1R signaling. The research shows that the ATRAP/Atrap expression can be altered in various pathological states in mouse (19, 20, 21, 22, 23) and human (10, 24, 25). For example, the Atrap protein degrades in response to the Ang II stimulation in various cells and organs, probably through the proteasome pathway (21, 26, 27). However, ATRAP protein does not have a lysine residue in the putative ubiquitination site. In addition, miR-376a and miR-135a act on and repress rat Atrap mRNA in neurons (28). However, these two miRNAs are expressed specifically in neurons and are not found in kidney tissue.

miRNAs are 21∼23 nucleotide noncoding RNAs that post-transcriptionally repress hundreds of target mRNAs. Drosha and Dicer are the miRNA-processing enzymes that are required for the maturation of miRNAs. After Drosha- and Dicer-mediated processing, miRNAs are loaded into the Argonaute (AGO) family of proteins, the active part of the RNA-induced silencing complex, binding the target mRNA strand complementary in the cytoplasm, inducing mRNA exoribonucleolytic degradation and translational repression (29, 30, 31). As enhancement of ATRAP/Atrap can repress pathogenic activation of the Ang II–AT1R signaling, the identification of evolutionarily conserved miRNAs acting on ATRAP/Atrap would be intriguing to define new therapeutic target of the Ang II–AT1R signaling. Consistent with this view, numerous miRNAs have been identified as the Ang II–AT1R signaling–associated disease phenotype modulators (32, 33, 34). Among these miRNAs, the miR-125 family plays critical roles in the growth, development, and incidence of cardiovascular diseases as well as various cancers (35, 36, 37).

In this study, we hypothesized that miRNA plays a role in regulating Atrap expression following Ang II treatment in renal tubular cells, and that this regulation is evolutionarily conserved. Our analysis revealed that miR-125a-5p and miR-125b-5p can directly repress both Atrap and ATRAP. Furthermore, inhibition of miR-125a-5p–miR-125b-5p resulted in the suppression of Ang II–AT1R signaling activation in mouse distal convoluted tubule (mDCT) cells. Our results should provide new insights into potential approaches for achieving organ-protective effects by repressing the miR-125 family associated with enhancing ATRAP/Atrap expression.

## Results

### Cloning, characterization, and the Ang II stimulation of mDCT cells

**Figure 1:**
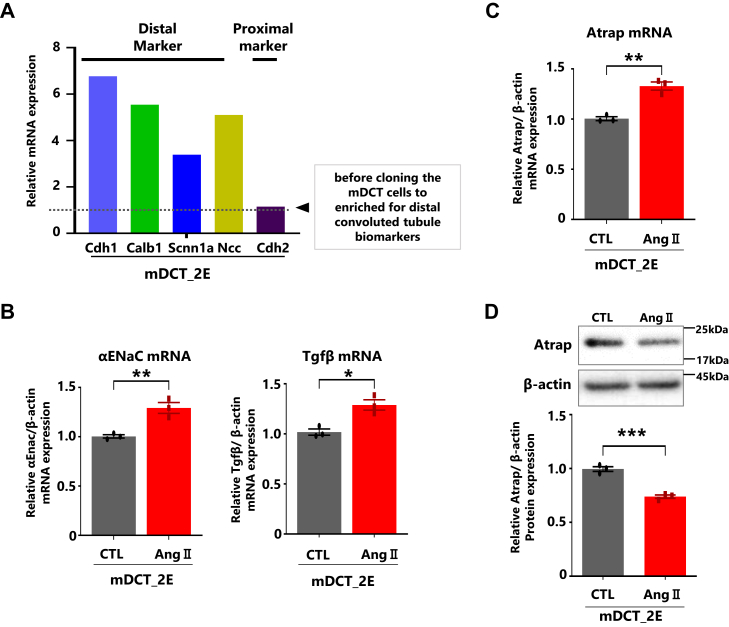
**Cloning,characterization, and the Ang II stimulation to the mouse distal convoluted tubular (mDCT) cells.** Comparison of mRNA expression levels of distal and proximal tubular markers in the mDCT_2E cells, and the response to Ang II treatment after 6 h. *A*, the relative mRNA levels of distal and proximal tubular markers in mDCT_2E cells as determined by RT–qPCR, normalized to β-actin. The mRNA levels in mDCT cells before cloning were set to 1 (n = 1). *B*, reactivity of Tgfβ and αENaC, the downstream gene of the AT1R signaling. The relative mRNA levels of αENaC and TGFβ were determined by RT–qPCR, normalized to β-actin (n = 3). The mRNA levels obtained without Ang II (control; CTL) were set to 1. *C*, reactivity of Atrap mRNA. The relative mRNA levels of Atrap were determined by RT–qPCR, normalized to β-actin (n = 3). The mRNA levels obtained without Ang II (CTL) were set to 1. *D*, reactivity of protein. The relative protein levels by Western blotting analysis, normalized to β-actin (n = 3). The protein levels obtained without Ang II (CTL) were set to 1. Data were obtained with three biologically independent experiments except (*A*). Values represent the means ± standard error. ∗*p* < 0.05, ∗∗∗*p* < 0.001 *versus* CTL (Ang II 0 μM) group. Data were analyzed *via* the unpaired *t* test. All preprocessing original Western blot data are shown in Fig. S5. αENaC, alpha epithelial sodium channel; Ang II, angiotensin II; AT1R, Ang II type 1 receptor; Atrap, mouse Atrap; qPCR, quantitative PCR; TGFβ, transforming growth factor beta.

ATRAP/Atrap is highly expressed in renal tissues, especially in the proximal and distal tubules (10). In addition, the inhibitory role of Atrap for the pathological Ang II–AT1R signaling is investigated in a mouse model (15). Therefore, we plan to analyze the regulatory mechanisms of Atrap in mDCT cells. Due to the heterogeneity and instability of the cell phenotype, we have cloned and characterized mDCT cells (10, 38). We selected mDCT_clone 2E because it showed higher expression of distal convoluted tubular cell markers (Figs. 1*A* and S1, *A*–*D*). From now on, we will refer to it as “mDCT.” We then observed the response of the Ang II treatment for 6 h. Consistent with previous reports (10), the mDCT cells showed sensitivity to Ang II, which resulted in increased expression of αENaC (alpha epithelial sodium channel) and transforming growth factor beta (TGFβ) mRNAs, both downstream targets of AT1R signaling. In addition, they increased the expression of Atrap mRNA (as seen in Fig. 1, *B* and *C*) but decreased the expression of Atrap protein in these cells (as observed in Figs. 1*D*, S1*E*, and S5).

### miRNAs repress the expression of Atrap in mDCT cells

**Figure 2:**
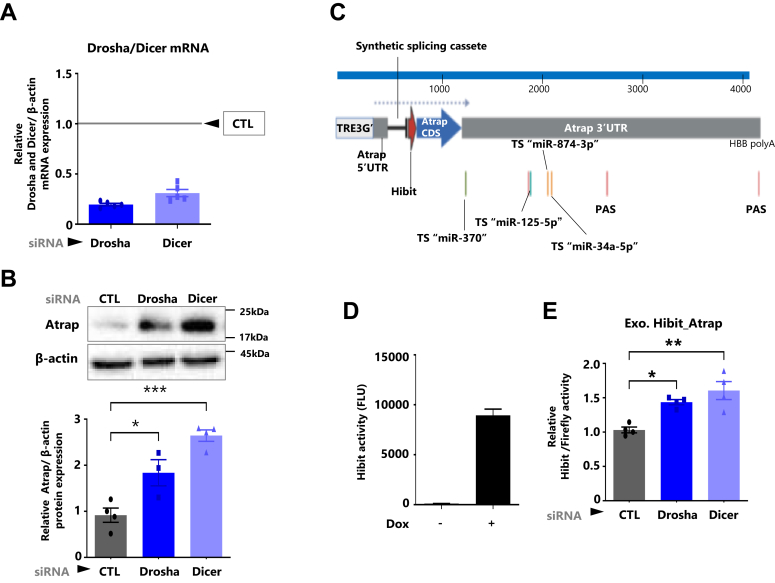
**miRNAs repress the expression of Atrap in mDCT cells.** Effect of the inactivation of mRNA pathway, using siRNAs of Drosha or Dicer, miRNA processing factors, and the analysis of endogenous/exogenous Atrap expression in mDCT cells. The mDCT cells were treated with negative control siRNA (control; CTL), Drosha/Dicer siRNA (Drosha/Dicer) for 48 h. *A*, confirmation of Drosha/Dicer mRNA expression by RT–qPCR, normalized to β-actin. The mRNA levels of the CTL group were set to 1 (n = 4–5). *B*, the relative protein expression of Atrap in mDCT cells was determined by Western blot analysis, normalized to β-actin expression. The protein levels of the CTL group were set to 1 (n = 3–4). *C* and *D*, establishment of mDCT cells capable of doxycycline (Dox)-induced Hibit-Atrap expression (schema created at biorender.com). Hibit-Atrap contains the 3′ UTR sequence encompassing even the most distant poly A site (PAS). *C*, confirmation of the induction of Hibit-Atrap with Dox treatment. Hibit activity was measured by a plate reader. Hibit activity levels of the Dox group were set to 1 (n = 2). *D*, the mDCT_Hibit-Atrap gene cells were treated with negative control siRNA (control; CTL), Drosha/Dicer siRNA (Drosha/Dicer) for 48 h, followed with treatment by Dox for 8 h, then dual luciferase reporter assay was performed. The Hibit activity was measured by a plate reader and normalized to firefly activity. The relative Hibit/firefly levels of the CTL group were set to 1 (n = 4). Data were obtained with three to five biologically independent experiments. Values represent the means ± standard error. ∗*p* < 0.05, ∗∗*p* < 0.01, ∗∗∗*p* < 0.001 *versus* siRNA-CTL group. Data were analyzed by (*B* and *E*) one-way ANOVA with Tukey’s post hoc test. The data shown are presented as the mean ± SEM. All preprocessing original Western blot data are shown in Fig. S5. Atrap, mouse Atrap; mDCT, mouse distal convoluted tubule cell; qPCR, quantitative PCR.

In mDCT cells, Ang II–AT1R caused an increase in Atrap mRNA and a decrease in Atrap protein. This indicated the presence of post-transcriptional regulation of Atrap expression. To investigate this mechanism, we analyzed the general effect of miRNAs on endogenous Atrap expression by repressing miRNAs with siRNA-mediated Drosha and Dicer knockdown (31). Knockdown of Drosha or Dicer was validated by RT–quantitative PCR (qPCR; Fig. 2*A*). And an increase in endogenous Atrap protein expression was observed (Figs. 2*B*, S2*A*, and S5). Next, we developed an mDCT cell line expressing the exogenous Hibit (a split Nanoluc fragment)-tagged Atrap gene under the doxycycline (Dox)-dependent exogenous promoter (mDCT_Hibit-Atrap gene) (39) (Fig. 2, *C* and *D*). Using this cell line, we investigated the post-transcriptional effects of Drosha knockdown nor Dicer knockdown. Our results showed that the knockdown of either Drosha or Dicer resulted in an increase in Hibit-Atrap expression transcribed from an exogenous promoter, for a duration of 8 h (Fig. 2*E*). These results indicated that miRNAs have the potential to repress Atrap protein expression in mDCT cells.

### Identification of miR-125a-5p/miR-125b-5p as an evolutionarily conserved direct repressor of Atrap expression

**Figure 3:**
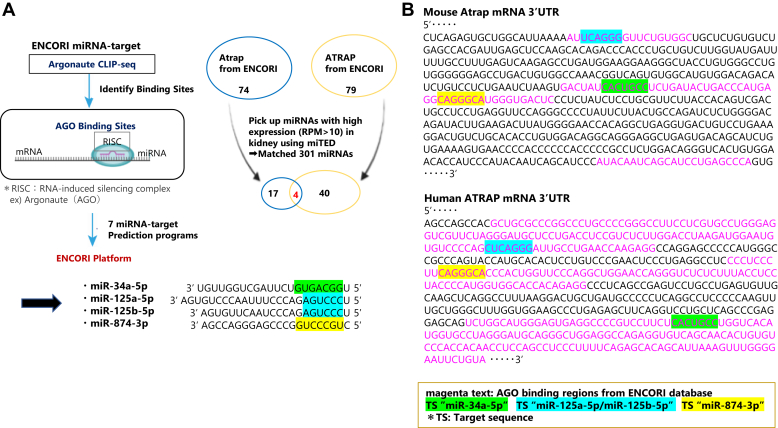
**Predicted the candidatesof miRNA targeting Atrap/ATRAP using the database.***A*, predicted the miRNAs targeting Atrap and ATRAP using ENCORI miRNA-target. ENCORI provides a comprehensive Ago2-mRNA binding dataset and includes both human and mouse genes. Then selected miRNAs with high expression (RPM >10) in kidney using miTED. Eventually, identified four candidate miRNAs, miR-34a-5p, miR-125a-5p, miR-125b-5p, and miR-874-3p. The sequence of each miRNA is shown, and the *shaded areas* indicate the binding site. This figure was created using biorender.com. *B*, represents fragments of Atrap and ATRAP mRNA 3′UTRs. *Magentaletters* indicate argonaute-binding sites. The *shaded areas* indicate target sequences of the corresponding miRNAs. Ago2, argonaute 2; ATRAP, human Atrap; Atrap, mouse Atrap.

To identify specific miRNAs that act on both Atrap and ATRAP mRNAs and are abundantly expressed in the kidney, we examined the predicted miRNAs using two databases, ENCORI (40) and miTED (41) (Fig. 3*A*, see *Experimental procedures* section in detail). As a result, we identified four candidate miRNAs: miR-34a-5p, miR-125a-5p, miR-125b-5p, and miR-874-3p, which may act on Atrap–ATRAP mRNA in renal tubules (Fig. 3*A*). The previously reported miRNAs targeting rat Atrap mRNA were excluded as candidates because miR-135a and miR-376a are less expressed in the kidney, and miR-370, which targets mouse Atrap mRNA, has no target site in ATRAP mRNA (28, 42).

**Figure 4:**
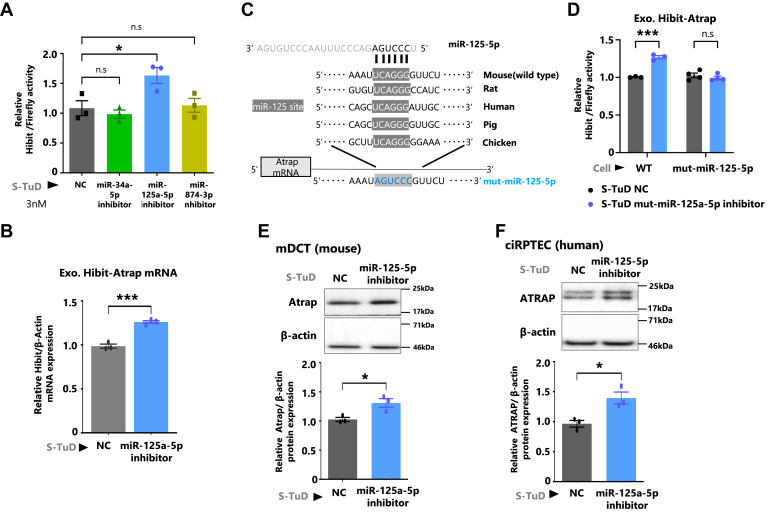
**Identification of miR-125-5p as an evolutionarily conserved direct repressor of Atrap–ATRAP expression.***A*, effect of the four candidate miRNAs on Atrap mRNA, employing synthetic tough decoy (S-TuD), an miRNA inhibitor. *C* and *D*, effect of the miR-125-5p on Atrap mRNA directly, by introducing the mutation to replace a complementary sequence at the site where miR-125a-5p–miR-125b-5p can bind. *E* and *F*, effect of the miR-125-5p inhibition on endogenous Atrap–ATRAP. *A*, the mDCT_Hibit-Atrap gene cells were treated with S-TuD (3 nM), negative control (NC), mmu-miR-34a-5p, mmu-miR-125a-5p, and mmu-miR-874-3p for 48 h, followed by the treatment with Dox for 8 h, then Dual-Luciferase Reporter Assay was performed. The Hibit activity was measured by a plate reader and normalized to firefly activity. The relative Hibit/firefly levels of the S-TuD NC group were set to 1 (n = 3). *B*, the mDCT_Hibit-Atrap gene cells were treated with S-TuD (3 nM), NC, and mmu-miR-125a-5p for 48 h, followed by the treatment with Dox for 8 h. The relative mRNA expression of Hibit-Atrap was determined by RT–qPCR, normalized to β-actin expression. The mRNA levels of the NC–CTL group were set to 1 (n = 3). *C*, represents miR-125a-5p sequences and Atrap–ATRAP mRNA 3′UTR sequences of various species (mouse, rat, chicken, pig, and human). The *shaded region* indicates a possible binding sequence. The mut-miR-125-5p refers to the sequence with mutation replacing the complementary base. *D*, the mDCT_Hibit-Atrap gene (WT) cells and the mDCT_mut-Hibit-Atrap gene (mut-miR-125-5p) cells were treated with S-TuD (3 nM), NC, and mmu-miR-125a-5p (miR-125-5p) for 48 h, followed by the treatment with Dox for 8 h, and then Dual-Luciferase Reporter Assay was performed. The Hibit activity was measured by a plate reader and normalized to firefly activity. The relative Hibit/firefly levels of both S-TuD NC groups were set to 1 (n = 3). *E* and *F*, the mDCT cells and human immortalized proximal tubular cells (ciRPTEC) were treated with S-TuD (3 nM), NC, and mmu-miR-125a-5p (miR-125-5p) for 48 h. The relative protein expression of Atrap–ATRAP in the mDCT cells and ciRPTEC was determined by Western blot analysis, normalized to β-actin expression. The protein levels of the NC group were set to 1 (n = 3). Data were obtained with three biologically independent experiments. *A*, one-way ANOVA with Tukey’s post hoc test: ∗*p* < 0.05 *versus* NC (n = 3). *B*, an unpaired *t* test: ∗*p* < 0.05 *versus* S-TuD NC (n =3). The data shown are presented as the mean ± SEM. *D*, values represent the means ± standard error. ∗∗∗*p* < 0.001 *versus* CTL group. Data were analyzed by two-way ANOVA with Tukey’s post-hoc test, n = 3 to 4. The data shown are presented as the mean ± SEM. *E* and *F*, values represent the means ± standard error. ∗*p* < 0.05 *versus* NC group. Data were analyzed by unpaired *t* test, n = 3. The data shown are presented as the mean ± SEM. All preprocessing original Western blot data are shown in Fig. S5. ATRAP, human ATRAP; Atrap, mouse Atrap; CTL, control; Dox, doxycycline; mDCT, mouse distal convoluted tubule cell; qPCR, quantitative PCR.

To determine the effect of the four candidate miRNAs on Atrap mRNA, we employed synthetic tough decoy (S-TuD), an miRNA inhibitor (Fig. S2*B*) (43). As shown in Figure 4*A*, only the miR-125a-5p inhibitor was able to enhance the Hibit-Atrap protein expression in mDCT_Hibit-Atrap gene cells (Fig. 4*A*). Note that the miR-125a-5p inhibitor could not discriminate between miR-125a-5p and miR-125b-5p, thus inhibiting both miRNAs. In addition, the miR-125a-5p inhibitor was found to increase the expression of exogenous Hibit-Atrap mRNA (Fig. 4*B*). These results indicated that miR-125a-5p–miR-125b-5p can repress both Atrap mRNA and protein expression at the post-transcriptional mRNA level. Furthermore, the inhibitors of miR-34a-5p and miR-874-3p did not increase Atrap expression. However, a single gene is regulated by multiple miRNAs (29, 30), thus miR-34a-5p and miR-874-3p might act in different cells/tissues and conditions.

Next, we analyzed whether miR-125a-5p/miR-125b-5p can directly repress Atrap expression or not. For this purpose, we introduced a point mutation of the miR-125a-5p/miR-125b-5p targeting sequence, which was replaced by a complementary sequence, into the Hibit-Atrap reporter gene and stably expressed it in mDCT cells (mDCT_mut-Hibit-Atrap gene) (Fig. 4*C*). We then evaluated the effects of the miR-125a-5p inhibitor on mut-Hibit-Atrap expression. Our results indicated that the mutation of miR-125a-5p–miR-125b-5p targeting sequence became insensitive to the miR-125a-5p inhibitor (Fig. 4*D*). This suggested that miR-125a-5p/miR-125b-5p directly acted on Atrap mRNA and repressed its expression.

To validate the evolutionarily conserved effect of the miR-125a-5p inhibitor on the expression of endogenous Atrap and ATRAP, we transfected the miR-125a-5p inhibitor into mDCT cells or human cloned immortalized renal proximal tubule epithelial cells (ciRPTECs) (24). The results showed that the miR-125a-5p inhibitor enhanced endogenous Atrap/ATRAP protein expression in both mDCT cells and ciRPTECs (Figs. 4, *E* and *F*, S2, *D* and *E*, and S5). Taken together, these results indicated that miR-125a-5p/miR125b-5p-mediated Atrap/ATRAP repression is evolutionarily conserved between mouse and human.

### Ang II promoted Atrap mRNA accumulation by repressing miR-125a-5p/miR125b-5p expression

**Figure 5:**
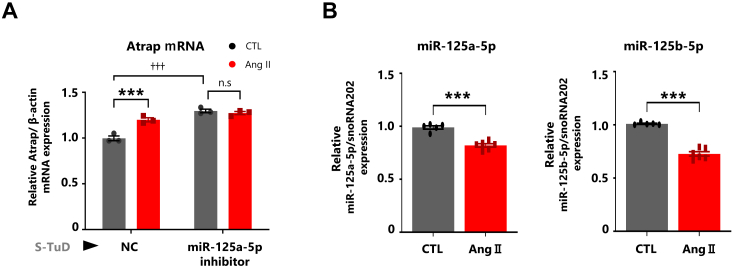
**Ang II promoted Atrap mRNA accumulation by repressing miR-125a-5p–miR125b-5p expression.** Effect of the miR-125-5p inhibition on Atrap mRNA in Ang II-stimulated mDCT cells. *A*, the relative mRNA expression of Atrap was determined by RT–qPCR, normalized to β-actin expression. The mRNA levels of the NC–CTL group were set to 1 (n = 3). *B*, the mDCT_Hibit-Atrap gene cells were treated with 1 μM Ang II for 6 h. The relative miRNA expression of miR-125a-5p and miR-125b-5p was determined by RT–qPCR analysis, normalized to snoRNA202 expression. The miRNA levels of the CTL group were set to 1 (n = 5–6). ∗*p* < 0.05, ∗∗*p* < 0.01, ∗∗∗*p* < 0.001 *versus* CTL. ^††^*p* < 0.01, ^†††^*p* < 0.001 *versus* NC–CTL. Data were obtained with three (*A*) or five to six (*B*) biologically independent experiments. Data were analyzed by (*A*) two-way ANOVA with Tukey’s post hoc test and (*B*) unpaired *t* test. The data shown are presented as the mean ± SEM. Ang II, angiotensin II; Atrap, mouse Atrap; CTL, control; mDCT, mouse distal convoluted tubule cell; NC, negative control; qPCR, quantitative PCR.

After observing an increase in Atrap mRNA and a decrease in Atrap protein in Ang II-treated mDCT cells (Fig. 1*C*), we examined the effect of miR-125a-5p/miR-125b-5p inhibition. To analyze this, we transfected the miR-125a-5p inhibitor into mDCT cells for 48 h, followed by treatment with Ang II for 6 h. The results showed that the miR-125a-5p inhibitor increased Atrap mRNA expression to levels similar to those induced by Ang II. No further Ang II-induced increase in Atrap mRNA was observed in the miR-125a-5p inhibitor–transfected condition (Fig. 5*A*). This suggested the possibility that the Ang II-induced Atrap mRNA enhancement was modulated by miR-125a-5p/miR-125b-5p. In this scenario, miR-125a-5p/miR-125b-5p needs to be repressed in the Ang II-treated condition. Consistent with this hypothesis, the expression of miR-125a-5p and miR-125b-5p was found to decrease in response to Ang II treatment (Fig. 5*B*). These results suggested that miR-125a-5p/miR125b-5p kept decreasing Atrap mRNA under normal conditions and that Ang II stimulation repressed miR-125a-5p/miR125b-5p to promote Atrap mRNA accumulation.

### Ang II promoted Atrap protein decrease by enhancing proteasome subunit expression

**Figure 6:**
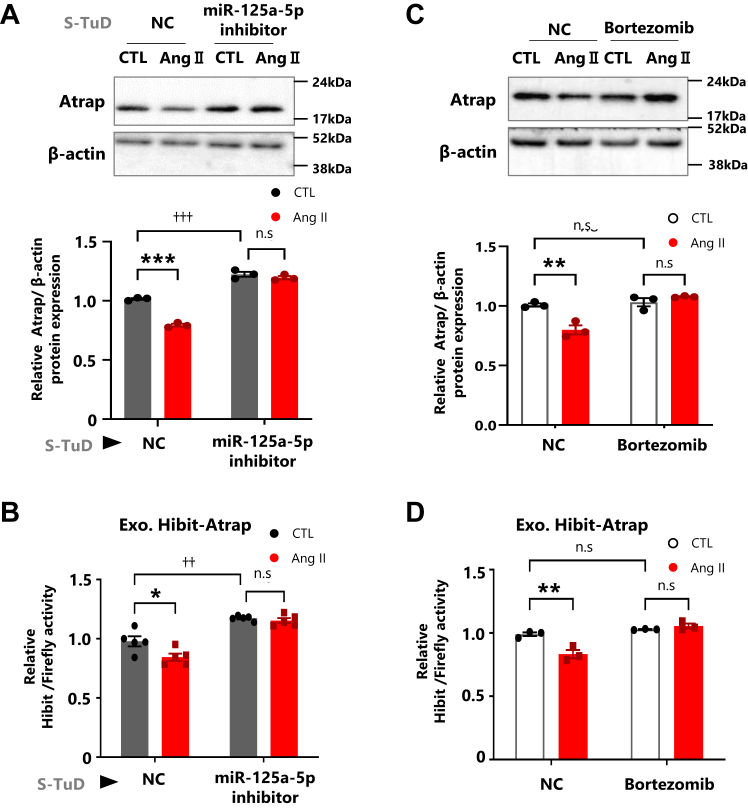
**Ang II enhanced proteasome subunit expression, reducing Atrap protein.***A* and *B*, effect of the miR-125-5p inhibition on Atrap mRNA in Ang II-stimulated mDCT cells. *C* and *D*, effects of the Ang II and bortezomib treatment on Atrap degradation in mDCT cells. *A*, the mDCT_Hibit-Atrap gene cells were treated with S-TuD (3 nM), negative control (NC), and mmu-miR-125a-5p (miR-125-5p) for 48 h, followed by the treatment with 1 μM Ang II for 6 h. The relative protein expression of Atrap was determined by the Western blot analysis, normalized to β-actin expression. The protein levels of the NC–CTL group were set to 1 (n = 3). *B*, the mDCT_Hibit-Atrap gene cells were treated with S-TuD (3 nM), NC, and mmu-miR-125a-5p (miR-125-5p) for 48 h, and then Dual-Luciferase Reporter Assay was performed after treatment with Dox and Ang II. Hibit activity was measured by a plate reader and normalized to firefly activity. The relative Hibit/firefly levels of the NC–CTL group were set to 1 (n = 5). *C*, the relative protein expression of Atrap, after 6 h of treatment with 1 μM Ang II and/or 10 nM bortezomib, was determined by Western blot analysis, normalized to β-actin expression. The protein levels of the NC–CTL group were set to 1 (n = 3). *D*, Dual-Luciferase Reporter Assay was performed after treatment with Dox and Ang II–bortezomib for 6 h. The Hibit activity was measured by a plate reader and normalized to firefly activity. The relative Hibit/firefly levels of NC–CTL group was set to 1 (n = 3). ∗*p* < 0.05, ∗∗*p* < 0.01, ∗∗∗*p* < 0.001 *versus* CTL. ^†^*p* < 0.05 *versus* NC–CTL. Data were obtained with three (*A*, *C*, and *D*) or five (*B*) biologically independent experiments. Data were analyzed by two-way ANOVA with Tukey’s post hoc test. The data shown here are presented as the mean ± SEM. All preprocessing original Western blot data are shown in Fig. S6. Ang II, angiotensin II; Atrap, mouse Atrap; CTL, control; Dox, doxycycline; mDCT, mouse distal convoluted tubule cell; S-TuD, synthetic tough decoy.

**Figure 7:**
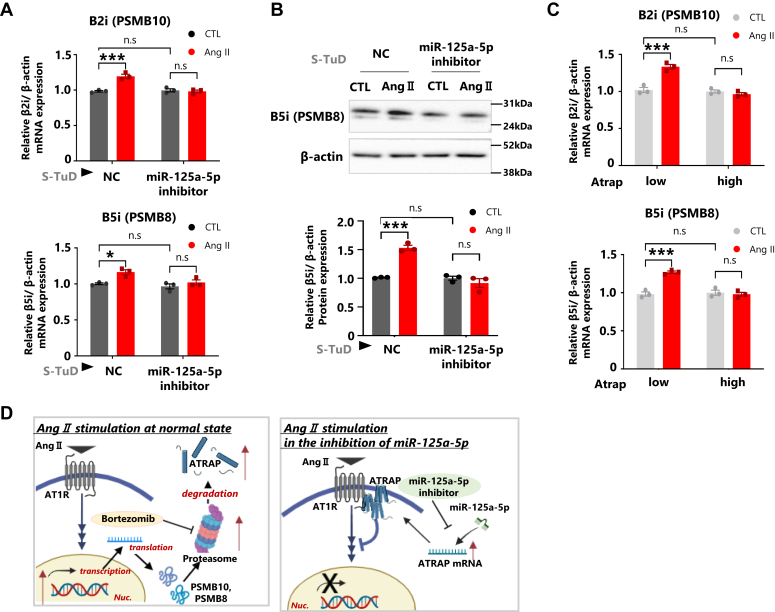
**Atrap upregulation and miR-125a-5p inhibition suppressed proteasome subunit expression stimulated by Ang II.***A* and *B*, effect of the miR-125-5p inhibition on Atrap mRNA in Ang II-stimulated mDCT cells. *C*, effects of the enhancing exogenous Atrap expression for the Ang II-induced proteasome subunit expression. *A*, the relative mRNA expression of β2i and β5i was determined by RT–qPCR, normalized to β-actin expression. The mRNA levels of the NC–CTL group were set to 1 (n = 3). *B*, the relative protein expression of β5i was determined by Western blot analysis, normalized to β-actin expression. The protein levels of the NC–CTL group were set to 1 (n = 3). *C*, the mDCT_Hibit-Atrap gene cells were treated with/without 3 μg Dox for 24 h, and then 1 μM Ang II was added for 6 h. The relative mRNA expression of β2i and β5i was determined by RT–qPCR, normalized to β-actin expression. The mRNA levels of the Dox (−)-CTL group were set to 1 (n = 3). *D*, a schematic diagram of Ang II–AT1R–proteasome pathway in our analysis, which was created at biorender.com. ∗*p* < 0.05, ∗∗*p* < 0.01, ∗∗∗*p* < 0.001 *versus* CTL. ^†^*p* < 0.05 *versus* NC–CTL. Data were obtained with three biologically independent experiments. Data were analyzed by two-way ANOVA with Tukey’s post hoc test. The data shown here are presented as the mean ± SEM. All preprocessing original Western blot data are shown in Fig. S7. Ang II, angiotensin II; Atrap, mouse Atrap; CTL, control; Dox, doxycycline; mDCT, mouse distal convoluted tubule cell; NC, negative control; qPCR, quantitative PCR.

As noted previously, however, Ang II treatment would decrease Atrap protein (Figs.6, *A* and *B*, S3*B*, and S6). This discrepancy may reflect the alternative regulatory mechanism for the decrease in Atrap protein in response to Ang II. Two reports described that the Ang II-induced downregulation of Atrap protein was promoted by transcriptional activation of the proteasome subunits β5i and β2i (Fig. 7*D*) (26, 27). To validate this possibility, we examined the involvement of proteolysis by the activation of the proteasome pathway using bortezomib, a protease inhibitor. The results showed that the Ang II-induced decrease in both endogenous Atrap protein and exogenous Hibit-Atrap protein was abolished by bortezomib treatment in mDCT cells (Figs. 6, *C* and *D*, S3*D*, and S6). Consistent with previous reports, the Ang II-stimulated upregulation of β5i and β2i, but not β1i, proteasome subunit mRNAs was observed as putative mechanism for the decrease in Atrap protein (Figs. 7*A* and S4*A*). Enhancement of β5i protein expression was confirmed by Western blotting (Figs. 7*B*, S4, *B* and *C*, and S7). In contrast to miR-125a-5p/miR-125b-5p inhibition, proteasome repression did not enhance Atrap protein expression under normal conditions (Fig. 6*C*, *upper panel*, lanes 1 and 3; and *bottom graph*, columns 1 and 3; and Fig. 6*D* columns 1 and 3). This indicated that the proteasome degraded Atrap protein only in response to Ang II but not under normal conditions.

### The miR-125a-5p inhibitor and Atrap enhancement repressed the Ang II-stimulating expression of the proteasome subunit

We next analyzed the involvement of miR-125a-5p/miR125b-5p in Atrap protein expression. Consistent with the previous results (Fig. 4), the miR-125a-5p inhibitor increased Atrap protein expression under normal conditions (Fig. 6*A*, *upper panel*, lanes 1 and 3; and *bottom graph*, columns 1 and 3). Unexpectedly, however, the miR-125a-5p inhibitor could also abolish the Ang II-induced Atrap protein repression (Fig. 6*A*, *upper panel*, lanes 3 and 4; and *bottom graph*, columns 3 and 4). The transcriptional regulation–independent effect of the miR-125a-5p inhibitor on Atrap protein expression was confirmed by using the Hibit-Atrap gene reporter, in which similar results of endogenous Atrap protein were observed (Fig. 6*B*). These results suggest that the miR-125a-5p inhibitor has the ability to suppress Ang II-induced proteasome activation.

Since Ang II stimulates the expression of proteasome subunits (Fig. 7, *A*–*C*, columns 1 and 2), we analyzed the effect of the miR-125a-5p inhibitor on their expression. Intriguingly, the miR-125a-5p inhibitor could repress the Ang II-induced expression of proteasome subunit (Fig. 7, *A* and *B*, columns 3 and 4). This is probably through the enhancement of Atrap protein expression, as it could repress Ang II–AT1R signaling. To support this notion, we found that the enhancement of exogenous Atrap expression could also repress the Ang II-induced proteasome subunit expression (Fig. 7*C*, columns 3 and 4).

### The inhibition of miR-125-5p ameliorates Ang II–AT1R signaling by playing a role in regulating Atrap protein expression

**Figure 8:**
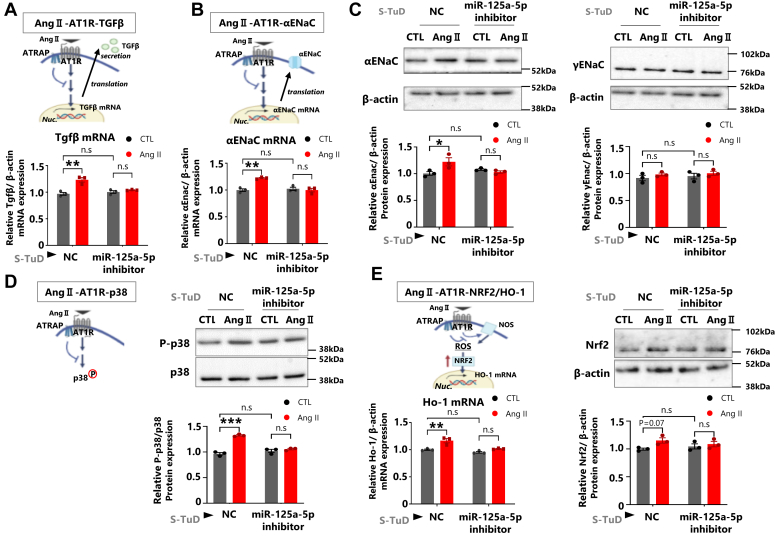
**The inhibition of miR-125-5p ameliorates Ang II–AT1R signaling by playing a role in regulating Atrap protein expression.** Effect of the miR-125-5p inhibition in response to various Ang II–AT1R signaling pathways in mDCT cells. *A*, *upper*, schematic diagram of Ang II–AT1R–TGFβ pathway in our analysis. *Lower*, the relative mRNA expression of Tgfβ as determined by RT–qPCR, normalized to β-actin expression. The mRNA levels of the NC–CTL group were set to 1 (n = 3). *B*, *upper*, schematic diagram of Ang II–AT1R–αENaC pathway in our analysis. *Lower*, the relative mRNA expression of αENaC was determined by RT–qPCR, normalized to β-actin expression. The mRNA levels of the NC–CTL group were set to 1 (n = 3). *C*, the relative protein expression of α/γENaC was determined by Western blot analysis, normalized to β-actin expression. The Western blot data of β-actin were the same as in Fig. S3*B* (*left panel*) and Figure 6*A* (*right panel*). The protein levels of the NC–CTL group were set to 1 (n = 3). *D*, *upper*, schematic diagram of Ang II–AT1R–p38 pathway in our analysis. *Lower*, the relative protein expression of p-p38 was determined by Western blot analysis, normalized to p38 expression. The protein levels of the NC–CTL group were set to 1 (n = 3). *E*, *upper*, schematic diagram of Ang II–AT1R–NRF2/HO-1 pathway in our analysis. *Lower*, the relative protein expression of Nrf2 was determined by Western blot analysis, and the relative mRNA expression of Ho-1 was determined by RT–qPCR, normalized to β-actin expression. The mRNA and protein levels of the NC–CTL group were set to 1 (n = 3). ∗*p* < 0.05, ∗∗*p* < 0.01, ∗∗∗*p* < 0.001 *versus* CTL. ^†^*p* < 0.05 *versus* NC–CTL. Data were obtained with three biologically independent experiments. Data were analyzed by two-way ANOVA with Tukey’s post hoc test. The data shown are presented as the mean ± SEM. All preprocessing original Western blot data are shown in Figs S8 and S9. αENaC, alpha epithelial sodium channel; Ang II, angiotensin II; AT1R, Ang II type 1 receptor; Atrap, mouse ATrap; CTL, control; HO-1, heme oxygenase 1; mDCT, mouse distal convoluted tubule cell; NC, negative control; NRF2, NFE2 like bZIP transcription factor 2; qPCR, quantitative PCR; TGFβ, transforming growth factor beta.

Finally, we examined the effect of miR-125a-5p inhibitor on other downstream effectors of Ang II–AT1R signaling; namely TGFβ mRNA (Fig. 8*A*), αENaC mRNA–protein (Figs. 8, *B* and *C*, S4*F*, and S8), phospho-p38 (Figs. 8*D*, S4*G*, and S8), and oxidative stress (NRF2 [NFE2 like bZIP transcription factor 2] protein and HO-1 mRNA) (Figs. 8*E*, S4*H*, and S9). We showed that the Ang II stimulation enhanced all these effectors of Ang II–AT1R pathway. However, transfection of the miR-125a-5p inhibitor decreased all these downstream effectors of Ang II–AT1R signaling (Figs. 8, *A*–*E*, S4, *F*–*H*, S8, and S9). No alternation in basal level of these effectors was observed by the miR-125a-5p inhibitor, although the basal level of Atrap protein expression is elevated (Figs. 8, *A*–*E*, S4, *F–H*, S8, and S9). γENaC protein (non–Ang II effector) showed no significant change neither by Ang II treatment nor by the treatment with miR-125a-5p inhibitor (Figs. 8*C*, S4*F*, and S8). These results showed that miR-125a-5p/miR-125b-5p inhibition not only enhanced Atrap protein expression but also decreased the Ang II–AT1R signaling pathway.

## Discussion

In the present study, we demonstrated that miR-125a-5p/miR-125b-5p directly acts on Atrap/ATRAP mRNA and represses its expression in mouse distal tubular cells, mDCT, and human proximal tubular epithelial cells, ciRPTEC. In addition, we found that miR-125a-5p/miR-125b-5p inhibition attenuated the proteasome-mediated decrease in Atrap protein expression in response to Ang II treatment. Furthermore, we revealed that miR-125a-5p–miR/125b-5p inhibition antagonized the effects of Ang II inducing cellular responses consisting of the proteasome subunits, TGFβ and αENaC expression, and p38 mitogen-activated protein kinase activation in mDCT cells. These results suggest that miR-125a-5p/miR-125b-5p promotes the Ang II–AT1R signaling and may be involved in the pathogenesis of hypertension and cardiovascular disease.

Consistently, a previous report presented that miR-125b-5p was identified as the driver of Ang II–TGFβ profibrotic signaling (37). In this report, the locked nucleic acid–based inhibition of miR-125b-5p was found to protect the Ang II infusion inducing cardiac fibrosis in mice. The authors showed that the Ang II infusion enhances miR-125b-5p expression through TGFβ signaling in the mouse heart. Furthermore, our group previously reported that Ang II infusion decreases Atrap mRNA and Atrap protein expression in the heart and outer renal medulla of mice (19, 44). On the other hand, in the present study, we showed that the Ang II treatment immediately decreased the expression of miR-125a-5p and miR-125b-5p in mDCT cells (Fig. 5*B*). The expression of miR-125a-5p/miR-125b-5p may vary because of time-dependent modulation, where primary Ang II signaling represses and secondary TGFβ signaling restores/promotes their expression, causing discrepancies in the effects on tissues and mDCT cells. Consistent with this view, TGFβ induces the transcriptional activation of the miR-125b-1 host gene (45). TGFβ–receptor expression may vary among cells/tissues, and therefore, the effect of Ang II stimulation on miR-125a-5p/miR-125b-5p expression would be different. Nevertheless, it remains coherent that the inhibition of miR-125a-5p and miR-125b-5p effectively suppresses Ang II-induced pathological responses in both the heart and kidney. Therefore, miR-125a-5p/miR-125b-5p could be a therapeutic target for hypertension and cardiac fibrosis.

Wang *et al.* (46) proposed that the Ang II-induced enhancement of proteasome subunits is prerequired to reduce Atrap expression in a mouse model. In line with this, our study demonstrated that the degradation of Atrap protein in mDCT cells by Ang II stimulation was mediated by the activation of proteasome subunits. This mechanism could be explained by our results in which the miR-125a-5p inhibitor caused the enhancement of exogenous Atrap expression or the increase in endogenous Atrap expression, thereby preventing the Ang II–AT1R signaling–mediated proteasome subunit expression (Fig. 7*D*).

On the other hand, we found that the miR-125a-5p inhibitors also upregulated ATRAP expression in human ciRPTEC cells (Fig. 4*F*). However, we were unable to analyze the effects of Ang II stimulation for ATRAP expression because of the faint Ang II response in our ciRPTEC cells. This is one of the limitations of our current work. We need to develop human cells that can respond to the Ang II stimulation. Alternative regulatory mechanisms may promote the decrease of ATRAP expression in human Ang II-associated pathologies (25) because the lysine residues of the Atrap ubiquitination site are missing in human ATRAP (Fig. S2*F*). Therefore, it is desirable to further investigate the role of the miR-125 family under Ang II stimulation in human tissues and cells.

We also observed that the miR-125a-5p inhibitor suppressed the activation of proteasome subunits (Fig. 7, *A* and *B*) and the degradation of Atrap protein (Fig. 5, *A* and *B*). However, miRNAs have multiple targets in a single signaling process (47). Therefore, our results may not be solely because of the increased Atrap expression. Although our study did not include an *in vivo* examination, such an analysis would be valuable to predict off-target effects for *in vivo* administration. In addition, it would be important to know the precise target organs/tissues for delivery of miR-125a-5p/miR-125b-5p inhibitor consisting of antisense locked nucleic acid or S-TuD to enhance its potential as a therapeutic target.

We showed that the Ang II treatment enhanced Atrap mRNA expression most likely through the decrease of miR-125a-5p/miR-125b-5p. This enhancement may be one of the feedback regulation mechanisms of Atrap protein abundance. In this case, Atrap protein would rapidly recover after Ang II ablations to suppress the pathogenic/excessive activation of Ang II–AT1R signaling (48). In addition to the post-transcriptional regulation of Atrap expression described in the present study, Atrap expression can also be transcriptionally regulated by Runx3 and USF1/USF2 (20, 49). Further analysis including these factors will help to understand the more precise regulatory mechanism of Atrap under Ang II-stimulated conditions.

The involvement of Ang II–AT1R signaling in the development and progression of nonalcoholic fatty liver disease and cancer has been well documented (50, 51). In the context of nonalcoholic fatty liver disease, ATRAP protein expression is decreased (22), whereas miR-125b-5p is upregulated (52). On the other hand, ATRAP expression is upregulated in various cancers, including bladder urothelial carcinoma, breast invasive carcinoma, hepatocellular carcinoma, lung adenocarcinoma, kidney cancer, and multiple gastrointestinal cancers (23). Intriguingly, the expression level of miR-125a-5p and/or miR-125b-5p is decreased in bladder urothelial carcinoma, breast cancer, hepatocellular carcinoma, colorectal cancer, cervical cancer, and lung adenocarcinoma (53, 54, 55, 56, 57, 58). Although the expression of the miR-125 family and ATRAP is negatively correlated, the direct relationship between them in these diseases remains unknown. Further investigation holds the potential to define new therapeutic targets.

In conclusion, our findings show that the miR-125a-5p/miR-125b-5p not only directly represses Atrap–ATRAP expression in renal tubular cells but also promotes Ang II–AT1R activation. These results suggest new avenues for potential therapeutic approaches to achieve the organ-protective effects of the miR-125a-5p/miR-125b-5p inhibitor in Ang II-associated diseases.

## Experimental procedures

### Cell culture

The mDCT cells were kindly provided by Dr Peter A. Friedman (University of Pittsburgh School of Medicine). These cells have been shown to have a phenotype of a polarized tight junction epithelium along with both morphological and functional features retained from the parental cells (59). The mDCT cells were cloned with limiting dilution, resulting in the isolation of six individual clones. These cloned mDCT cells, along with other noncloned mDCT cells, were maintained in Dulbecco’s modified Eagle’s medium (DMEM)/Ham's F12 medium supplemented with 5% fetal bovine serum (FBS) (Sigma–Aldrich) in a CO₂ incubator.

The normal human RPTECs were purchased from Lonza (catalog no.: CLCC-2553, lot no.: 0000203150, Caucasian female, 10 years old) and immortalized by infecting it with lentivirus-expressing human telomerase reverse transcriptase and shRNA targeting p16 (plenti6_TERT_sh-p16). The immortalized RPTECs (ciRPTECs) were cloned as mentioned previously (24). The ciRPTECs were cultured in DMEM with 10% FBS in a CO₂ incubator.

### Isolation of genomic DNA and cloning the complementary DNA of Atrap including the 3′UTR region

Mouse genomic DNA was extracted from mDCT cells. The Wizard Genomic DNA Purification Kit from Promega was used for this purpose, according to the manufacturer's instructions. The extracted DNA samples were then used as templates for PCR amplification of the target genes, specifically the Atrap gene with its 3′ UTR. The following primers were used: Atrap (forward: 5′-AGCTCTGTGAGCRRGTGGTC-3′, reverse: 5′-TAAAGGTGCCTCCCTCAGGA-3′).

### Predicted miRNAs targeting Atrap–ATRAP

To identify evolutionarily conserved miRNAs targeting Atrap–ATRAP mRNA, we employed two databases: ENCORI (40) and miTED (41). miRNAs typically act in the site where RNA-induced silencing complex, such as Argonaute1–4 (Ago1–4), binds to the target gene mRNAs. ENCORI provides a comprehensive Ago2–mRNA binding dataset (Fig. 3*A*) and includes both human and mouse genes. On the other hand, miTED provides valuable information on miRNA expression levels in various human tissues and cell lines. As a result, we identified four candidates according to the algorithm in Figure 3*A*.

### Establishment of mDCT cells capable of Dox-induced Hibit-mouse Atrap gene expression

In this study, we constructed three plasmids: pLenti_TetOn_Hibit-Atrap gene (Hibit-Atrap gene, containing blasticidin-resistance gene), mutation-miR-125-5p_binding_site-Hibit-Atrap gene (mut-miR-125-Hibit-Atrap gene, containing blasticidin-resistance gene), and pLenti_SV40p-Luc2 (SV40-Luc2, containing puromycin-resistance gene) plasmids. Dox was used to induce Hibit-Atrap based on the Tet-On system. The Hibit tag was inserted at the 5′-terminal region of the Atrap coding sequence. Detailed plasmid maps are available upon request. Lentiviral supernatants were produced as described elsewhere. Briefly, pLenti_TetOn_Hibit-Atrap gene (including mutant plasmid) or pLenti_SV40p-Luc2 (3 μg), pLP1, 3 μg of pLP2/VSVG (3 μg; Thermo Fisher Scientific), and pAdvantage (1.3 μg; Promega) were mixed in Opti-MEM medium (1.5 ml; Thermo Fisher Scientific) and added to Lipofectamine 2000 (39.9 μl; Thermo Fisher Scientific) in Opti-MEM (1.5 ml). The resulting solution was mixed and incubated for 20 min at room temperature. While incubating the DNA–Lipofectamine mixture, LentiX human embryonic kidney 293T cells (Thermo Fisher Scientific) (5 × 10^6^) were seeded in a poly-l-lysine-coated 10 cm tissue culture plate. After the incubation, the DNA–Lipofectamine mixtures were added to the LentiX human embryonic kidney 293T cells. At 8 h post-transfection, the medium was exchanged with DMEM containing 10% FBS and 10 μM forskolin. After 24∼48 h, the culture supernatants were collected and filtered through 0.22 μm Steriflip filters (Millipore) to generate the lentiviral supernatants. For the lentiviral infections, the lentiviral supernatant (2 ml) was incubated with mDCT cells or Hibit-Atrap gene (including mutant plasmid) mDCT (1.0 × 10^6^) for 24 h. Thereafter, the lentiviral supernatants were discarded followed by the addition of DMEM/Ham's F12 containing blasticidin (1.5 μg/ml) or puromycin (2.5 μg/ml). Finally, we stably cotransfected the mDCT cells with a plasmid expressing Hibit-Atrap and Luc2. The Hibit tag served as a NanoLuc reporter to Atrap protein expression, and Luc2 was also employed as a firefly luciferase to measure cellular internal standards, which allowed the use of the Dual-Luciferase Reporter Assay system.

### siRNA and cell transfection

The following siRNAs purchased from Qiagen were used: Drosha siRNA #1: (Mm_Etohi2_1 FlexiTube siRNA), Dicer siRNA #1: (Mm_Dicer1_1 FlexiTube siRNA) and AllStars negative control siRNA.

The mDCT cells were seeded in 12-well plates and transfected with Lipofectamine RNAiMax Reagent (Thermo Fisher Scientific) for 48 h at 37 °C according to the manufacturer’s protocol.

### miRNA inhibitor assay (S-TuD assay)

The S-TuD were obtained from Ajinomoto Bio-Pharma, including mmu-miR-34a-5p, mmu-miR-125a-5p, mmu-miR-874-3p, and a negative control.

Transfection of the S-TuD into mDCT cells/ciRPTEC was performed in a 12-well plate, seeded at a density of 5 × 10^4^/1 × 10^5^ cells per well and transfected with 75 μl of OptiMEM, 1 μl of Lipofectamine 2000 Reagent, and 1 or 3 μl of the respective S-TuD (mmu-miR-34a-5p, mmu-miR-125a-5p, mmu-miR-874-3p, or negative control). S-TuD was prepared at a concentration of 1 μM, and the final concentration used for transfection was 1 to 3 nM. After 48 h of incubation, S-TuD-treated cells were used in subsequent experiments.

### Dual-Luciferase Reporter Assay

The mDCT_Hibit-Atrap gene cells transfected with each S-TuD were seeded into a 96-well plate containing 100 μl of DMEM/Ham's F12 medium supplemented with 5% FBS. The cells were treated with or without Dox (3 μg/ml) and incubated for 4∼8 h. After incubation, half of the medium was removed from each well, and 50 μl of ONE-Glo EX Luciferase Assay Buffer (Promega) was added. The reaction was allowed to occur at room temperature for 10 min. Luminescence measurement was performed to quantify the Luc2 (firefly luciferase) signal using a plate reader. Subsequently, 50 μl of NanoDLR Stop & Glo Buffer containing 1% NanoDLR Stop & Glo Substrate and 1% Lgbit Protein was added to each well. Then again, the plate was incubated for 10 min at room temperature to allow the reaction. Luminescence measurement was performed to quantify the NanoLuc (Hibit + Lgbit) signal using a plate reader. This experimental setup helped to determine the quantification of Hibit-Atrap expression using the NanoLuc reporter (Hibit) and normalization with the internal standard Luc2 (firefly luciferase).

### Western blot analysis

Western blot analysis was performed as described elsewhere (60). Briefly, total protein was extracted from cells using a sample buffer containing SDS (1%). Then, the protein concentration in each sample was measured with a Qubit 2.0 Fluorometer (Thermo Fisher Scientific). An equal amount of each protein extract was resolved on a 5 to 20% polyacrylamide gel (ATTO Corporation) and electrophoresed. After separation, the proteins were transferred to a polyvinylidene fluoride membrane. The membranes were blocked for over 1 h at room temperature with Tris-buffered saline with Tween containing skim milk (5%) or Blocking One-P (nacalai tesque) and probed overnight at 4 °C with specific primary antibodies. Antibodies against the following proteins were used: Atrap and ATRAP (1:1000–3000 dilution, rabbit, developed in our previous study (60)), β-actin (1:5000 dilution, catalog no.: A5441, mouse, Sigma–Aldrich), β5 (PSMB5) (1:10,000 dilution, catalog no.: 19178-1-AP, rabbit, Proteintech), β5i (PSMB8) (1:1000 dilution, catalog no.: 13635, rabbit, CST), αENaC (1:1000 dilution, PA1-920A, rabbit, Invitrogen), γENaC (1:2000 dilution, catalog no.: ab3468, rabbit, abcam), active p38 (1:3000 dilution, catalog no.: V121A, rabbit, Promega), p38 (1:1000 dilution, N-20, rabbit, Santa Cruz), and Nrf2 (1:2000 dilution, catalog no.: GTX103322, rabbit, GeneTex). The membranes were washed and further incubated with an appropriate secondary antibody for 60 min at room temperature. When detecting Atrap–ATRAP, αENaC, γENaC and p38, anti-Rabbit immunoglobulin G (IgG), horseradish peroxidase (HRP)-Linked Whole Ab (NA934-1Ml, donkey, GE Healthcare), was diluted 1:2000 to 5000 with Tris-buffered saline with Tween containing skim milk (5%). When detecting β-actin, antimouse IgG, HRP-Linked Whole Ab (NA931-1Ml, sheep, GE Healthcare) was diluted 1:5000. When detecting Active p38, anti-Rabbit IgG, HRP-Linked Whole Ab (NA934-1Ml, donkey, GE Healthcare), was diluted 1:5000 with Signal Enhancer Hikari solution B (nacalai tesque). Full-range Rainbow Molecular Weight Markers (Cytiva) (Figure 6, Figure 7, Figure 8, *C*–*E*, S3, *B* and *D*, and S4, *B*, *C*, *F*, *G*, and *H*) and DynaMarker protein MultiColor Ladder Marker (Figure 1, Figure 2, Figure 4, *E* and *F*, S1*E*, and S2, *A*, *E*, and *F*), stable II were used as molecular weight markers. The bands were visualized using Luminata Classico/Forte (Merck) or ImmunoStar LD (Fujifilm Wako Pure Chemical) as the enhanced chemiluminescence substrate. The resulting images were quantitatively analyzed using a Chemidoc Touch Imager (Bio-Rad Laboratories). All these experiments were performed at least three times, and representative results are illustrated.

### RT–qPCR analysis

Total RNA was extracted from mDCT cells using the NucleoSpin RNA Plus Kit (Takara Bio) or MagMAX mirVana Total RNA Isolation Kit (Thermo Fisher Scientific), and complementary DNA was produced using ReverTra Ace qPCR RT Master Mix with genomic DNA Remover (Toyobo) and TaqMan MicroRNA Reverse Transcription Kit (Thermo Fisher Scientific). RT–qPCR was performed with a Bio-Rad CFX96 Touch Real-Time PCR Detection System (Bio-Rad Laboratories), and the reverse-transcribed products were incubated with THUNDERBIRD Next SYBR qPCR Mix (Toyobo) or TaqMan Fast Advanced Master Mix (Thermo Fisher Scientific). The Atrap-Ho-1 mRNA levels were normalized to those of β-actin. The miR-125a-5p and miR-125b-5p miRNA levels were normalized to those of snoRNA202.

The following primers were used: Atrap (forward: 5′-CCACCATCTTCCTGGACATT-3′, reverse: 5′-AGACGAGGCAGCAAGAGAAG-3′), Drosha (forward: 5′-GGACCATCACGAAGGACACT-3′, reverse: 5′-CACGGGTCTCTTGGTTTTGT-3′), Dicer (forward: 5′-ACCAAGTGATCCGTTTACGC-3′, reverse: 5′-CAACCGTACACTGTCCATCG-3′), β-actin (forward: 5′-GCCGCCAGCTCACCAT-3′, reverse: 5′-TCGTCGCCCACATAGGAATC-3′), Scnn1a (αENaC): (forward: 5′-ACCCCGTGAGTCTCAACATC-3′, reverse: 5′-CCTGGCGAGTGTAGGAAGAG-3′), Tgf-β (forward: 5′-TGCTTCAGCTCCACAGAGAA-3′, reverse: 5′-TGGTTGTAGAGGGCAAGGAC-3′). Cdh1 (cadherin; forward: 5′-AGCCATTGCCAAGTACATCC-3′, reverse: 5′-AAAGACCGGCTGGGTAAACT-3′), Cdh2 (forward: 5′-AGGGTGGACGTCATTGTAGC-3′, reverse: 5′-CTGTTGGGGTCTGTCAGGAT-3′), Calb1 (calbindin 1; forward: 5′-CCACCTGCAGTCATCTCTGA-3′, reverse: 5′-TTCCGGTGATAGCTCCAATC-3′), Aqp2 (aquaporin 2; forward: 5′-TTGCCATGTCTCCTTCCTTC-3′, reverse: 5′-GGTCAGGAAGAGCTCCACAG-3′), Ncc (sodium-chloride cotransporter; forward: 5′-CTGGAGAACCTGTTCGCTTC-3′, reverse: 5′-GATGTCACCATGACCGACAG-3′), Pth1r (parathyroid hormone 1rReceptor; forward: 5′-ATCTTCGTGAAGGACGCTGT-3′, reverse: 5′-CCCTCCACCAGAATCCAGTA-3′), At1r (forward: 5′-GGAAACAGCTTGGTGGTGAT-3′, reverse: 5′-ACATAGGTGATTGCCGAAGG-3′), Hibit-Atrap (forward: 5′-AGAAGATCAGCGGAGAGCTG-3′, reverse: 5′-GGCCAGGATAGTGAAGTTGC-3′), β1i (PSMB9) (forward: 5′-TCTTCTGTGCCCTCTCAGGT-3′, reverse: 5′-GGTCCCAGCCAGCTACTATG-3′), β2i (PSMB10) (forward: 5′-CTTTACTGCCCTTGGCTCTG-3′, reverse: 5′-GTGATCACACAGGCATCCAC-3′), β5i (PSMB8) (forward: 5′-CAGTCCTGAAGAGGCCTACG-3′, reverse: 5′-CACTTTCACCCAACCGTCTT-3′), and Ho-1 (forward: 5′-TTGAGGAGCTGCAGGTGATG-3′, reverse: 5′-TGCCAACAGGAAGCTGAGAG-3′). For the detection of miRNA expression, we purchased TaqMan MicroRNA Assay (has-miR-125a-5p, has-miR-125b-5p, and snoRNA202) from Thermo Fisher Scientific.

### Statistical analysis

Statistical analyses were performed with GraphPad Prism9 (GraphPad Software). All data are shown as the mean ± SEM. Differences were analyzed using the following statistical tests. Two-way ANOVA, followed by Tukey’s post hoc analysis, was performed to determine differences between CTL (control) and Ang II groups (Figs. 5, *A*–*C*, 6, 7, 8, and S4, *A*, *B*, and *D*). One-way ANOVA, followed by Tukey’s post hoc analysis, was performed to determine differences between CTL and other groups (Figs. 2, *B* and *E*, and 4*A*). In addition, an unpaired *t* test was used to analyze the differences between the two groups (Figs. 1, *B*–*D*, 4, *B*, *E*, and *F*, and 5*D*). *p* Values <0.05 were considered statistically significant. Data were obtained with three to five biologically independent experiments.

## Data availability

The data presented in this study are available on request from the corresponding author.

## Supporting information

This article contains supporting information (10, 15, 38, 59).

## Conflict of interest

The authors declare that they have no conflicts of interest with the contents of this article.
