# Supplementary material for: miR-125a-5p/miR-125b-5p contributes to pathological activation of angiotensin II-AT1R in mouse distal convoluted tubule cells by the suppression of Atrap
Source: J Biol Chem. 2023 Nov 21;299(12):105478. doi: 10.1016/j.jbc.2023.105478 (PMC10755798; doi:10.1016/j.jbc.2023.105478)

Supporting Information

Supporting Figure 1. Cloning, and characterization of mouse distal convoluted tubule (mDCT) cells.

A Comparison of mRNA expression levels of distal (Cdh1/E-cadherin, Calb1, Aqp2) and proximal tubule markers (Cdh2/N-cadherin) in mDCT cells, and **(B,C)** mRNA expression of the functional distal convoluted tubule markers (Scnn1a/αENaC, Ncc, and Pth1r) [10, 15, 38, 59], At1r and Atrap, in mDCT cells. The relative mRNA levels of Cdh2, Cdh1, Calb1, Aqp2, Scnn1a, Ncc, Pth1r, Atrap, and At1r in before and 6 cloned cells were determined by RT-qPCR, normalized to β-actin expression. mRNA levels of before-cloned mDCT cells were set to 1 (n=1). **D** Morphology of the mDCT before-cloned/2E cells is shown. Scale bar: 100 μm

E Biologically independent experimental data of Fig. 1D. The number represented in right of blot indicated the size (kDa) of molecular weight marker.

A-C Data were obtained with one biologically experiments.

Note that mDCT_cloned 2E cells displayed an epithelial-like cell morphology. Based on these results, we selected the clone_2E for further analysis in our study.

Supporting Figure 2. A schematic diagram of the strategy of miRNA inhibitor, S-TuD.

A Biologically independent experimental data of Fig. 2B. **B** A schematic diagram of S-TuD's mechanism. The RISC complex binds complementarily to the MBS, resulting in the inhibition of miRNA activity. **C** Effect of the four candidate miRNAs on Atrap mRNA, employing S-TuD at 1nM, negative control (NC), mmu-miR-34a-5p, mmu-miR-125a-5p, and mmu-miR-874-3p for 48 hours, followed by the treatment with Dox for 8 hours, then dual-luciferase reporter assay was performed. The result is the supporting data in Fig. 4A, which shows that S-TuD is effective even at 1 nM and the same results are observed under different conditions. The hibit activity was measured by a plate reader and normalized to firefly activity. The relative Hibit/firefly levels of the S-TuD NC group were set to 1. (n=3). **D** Biologically independent experimental data of Fig. 4E. **E** Biologically independent experimental data of Fig. 4F. **F**

Alignments of mouse Atrap and human ATRAP protein sequence. Note that ATRAP has no ubiquitination sites in contrast to Atrap. The sequence alignment was generated using bioedit. Data were obtained with three biologically independent experiments. Values represent the means±standard error. *p<0.05, **p<0.01, ***p<0.001 vs. S-TuD-NC group. Data were analyzed by one-way ANOVA with Turkey's post-hoc test. The data shown are presented as the mean ± SEM. The number represented in right of blot indicated the size (kDa) of molecular weight marker.

Supporting Figure 3. A schematic diagram of our experiments. A A schematic diagram of S-TuD transfection and Ang II treatment assay, created at biorender.com. **B** Biologically independent experimental data of Fig. 6A. The Western blot data of β-actin were the same as in Fig. 8C. **C** A schematic diagram of Ang II and Bortezomib treatment assay, created at biorender.com. **D** Biologically independent experimental data of Fig. 6C. The number represented in right of blot indicated the size (kDa) of molecular weight marker.

Supporting Figure 4

(A, B) The mDCT_Hibit-Atrap gene cells were treated with S-TuD (3nM), negative control (NC) and mmu-miR-125a-5p (miR-125-5p) for 48 hours, followed by the treatment of 1μM Ang II. (D) Effects of the enhancing exogenous Atrap expression for the Ang II-induced proteasome subunits expression. **A** The relative mRNA expression of β1i was determined by RT-qPCR, normalized to β-actin expression. The mRNA levels of the NC-CTL group were set to 1. (n=3). **B** The relative protein expression of β5 was determined by western blot analysis, normalized to β-actin expression. The protein levels of the NC-CTL group were set to 1. (n=3). **C** Biologically independent experimental data of Fig. 7B and Fig. S4B. **D** The mDCT_Hibit-Atrap gene cells were treated with/without 3μg doxycycline for 24 hours, then added 1μM Ang II for 6 hours. The relative mRNA expression of β1i was determined by RT-qPCR, normalized to β-actin expression. The mRNA levels of the Dox (-)-CTL group were

set to 1. (n=3). **E** A schematic diagram of Ang II and Dox treatment assay, created at biorender.com. **F-H** Biologically independent experimental data of Fig. 8C-E. The Western blot data of β-actin in Fig. S4F and S4H were the same as in Fig. 8E and 8C (left panel), respectively. All pre-processing original Western blot data were shown in Fig. S7. The number represented in right of blot indicated the size (kDa) of molecular weight marker.

A, B and **D** *$p < 0.05$, **$p < 0.01$, ***$p < 0.001$ vs CTL. † $p < 0.05$ vs NC-CTL. Data were obtained with three biologically independent experiments. Data were analyzed by two-way ANOVA with Turkey's post-hoc test. The data shown are presented as the mean ± SEM.

Supporting Figure 5-9. Each full sheet of Western blot data.

Full sheet of Western blot data of each experiment. The right- or left-most lane represents the data for the loading control. 0.5 and 1.5 times the standard amount was loaded. The areas enclosed by rectangles are those shown in the corresponding Figure. Red squares are shown in the main Figure and blue squares are shown in the Supporting Figure. The Western blot data of β-actin in Fig. S8 and S9 were the same as in Fig. S6 top. The number represented in right of blot indicated the size (kDa) of molecular weight marker.

Supporting Figure 1

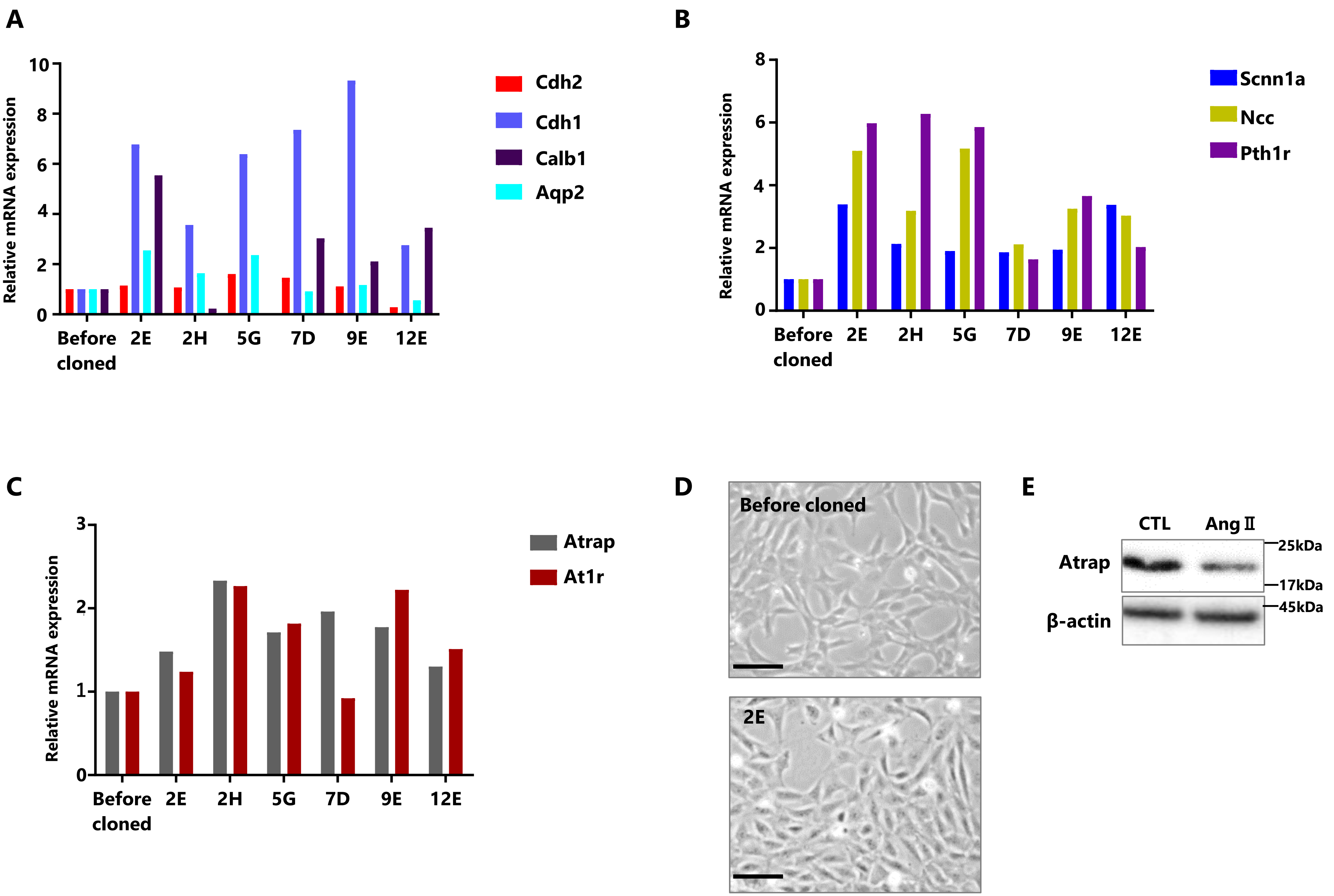

Supporting Figure 2

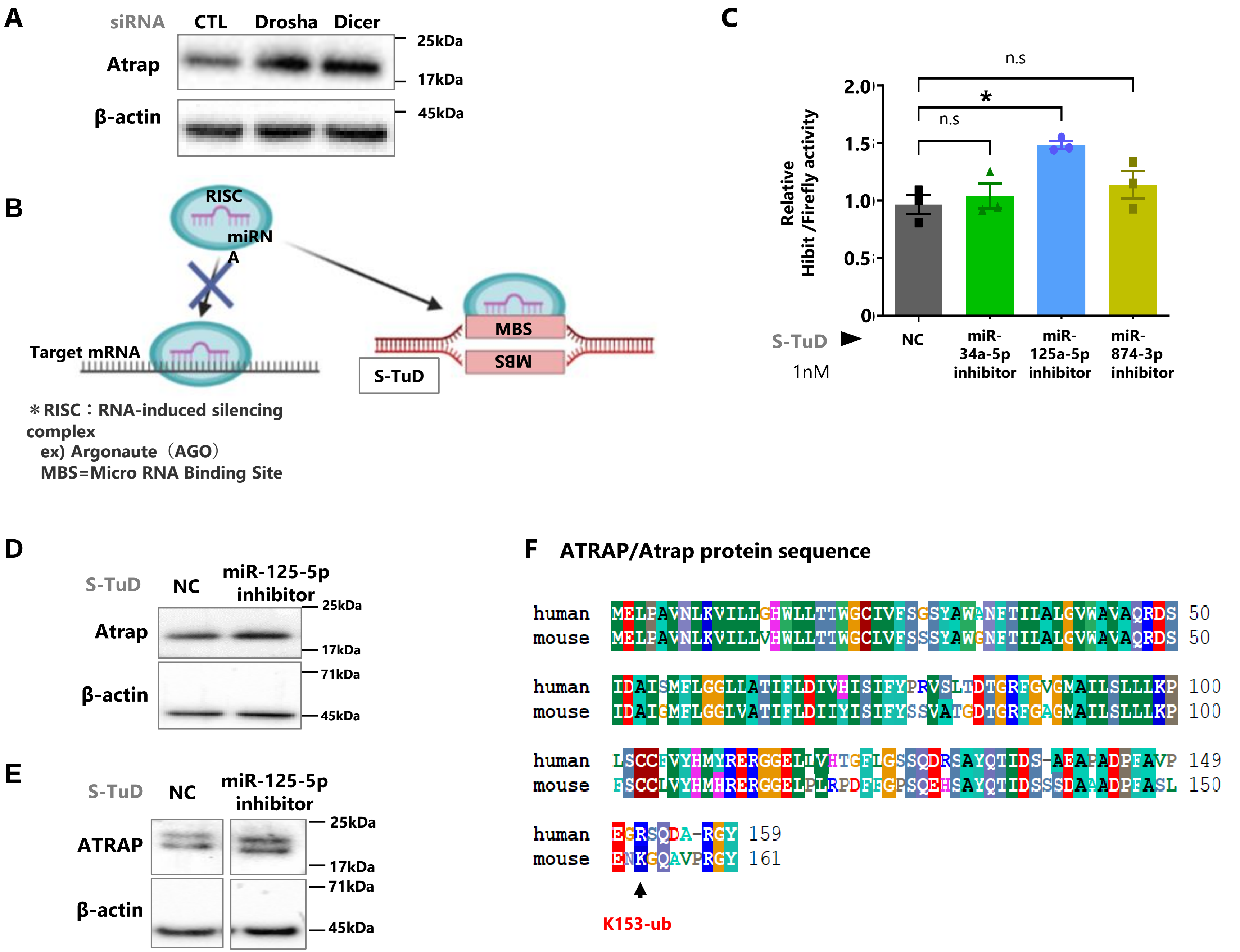

Supporting Figure 3

A **S-TuD transfection and Ang II treatment assay (Figure 6A, 6B, 7A, 7B, 8, S3B, S4A-C and S4F-H)**

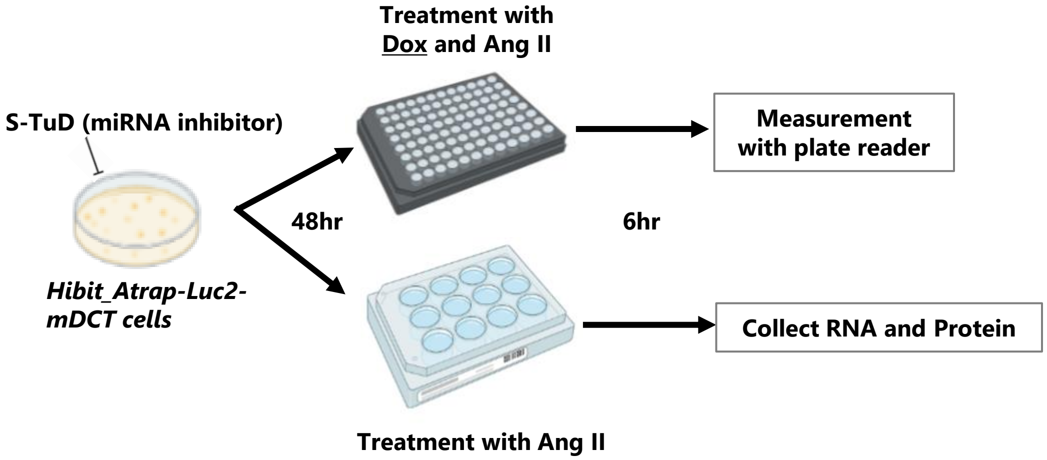

B

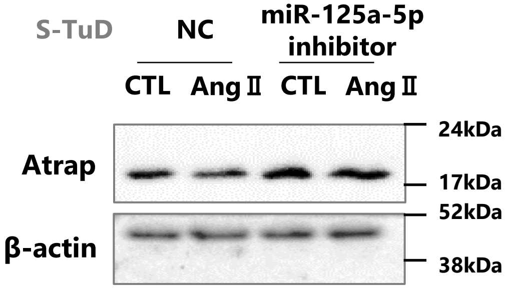

C **Ang II and Bortezomib treatment assay (Figure 6c, d)**

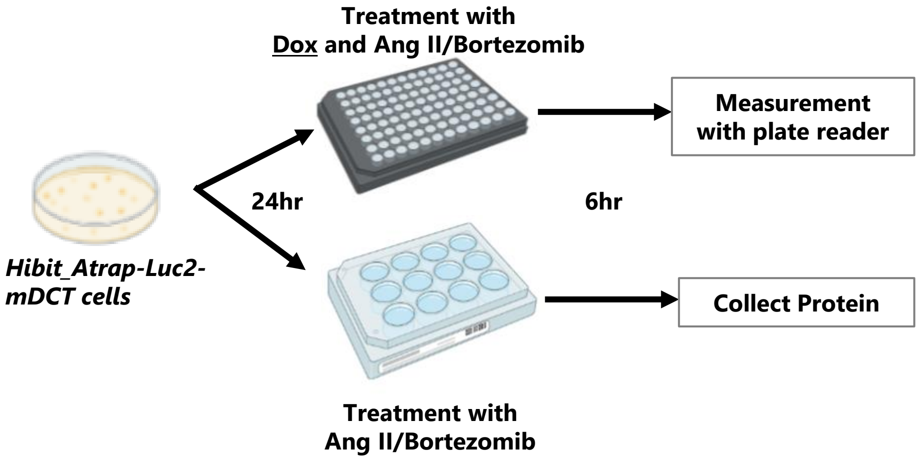

D

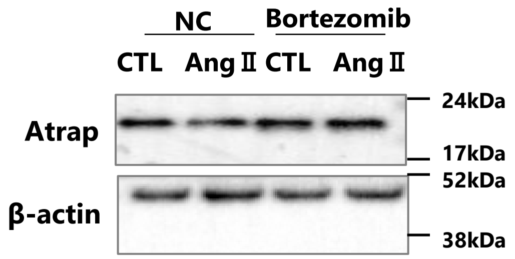

Supporting Figure 4

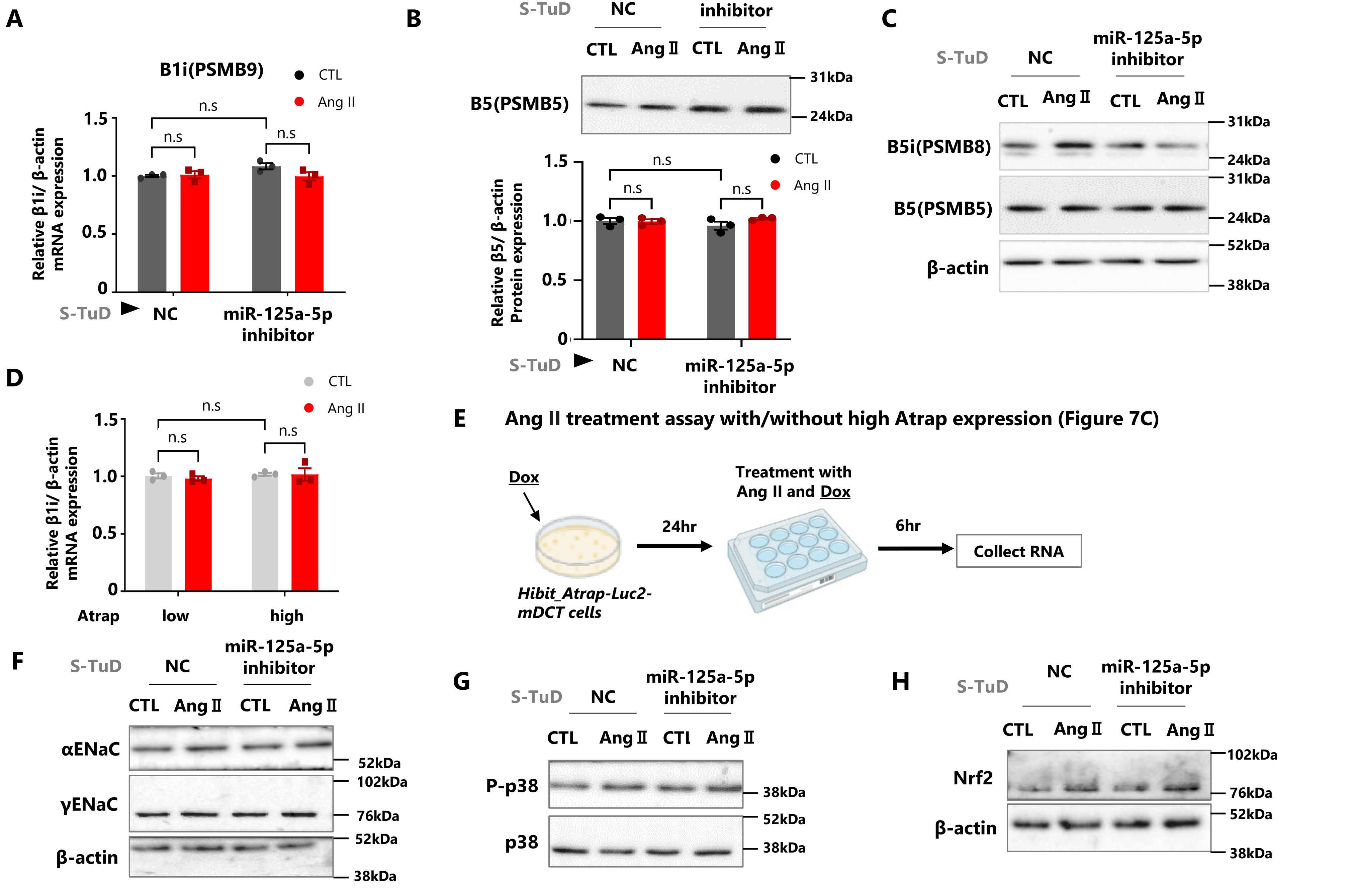

Supporting Figure 5

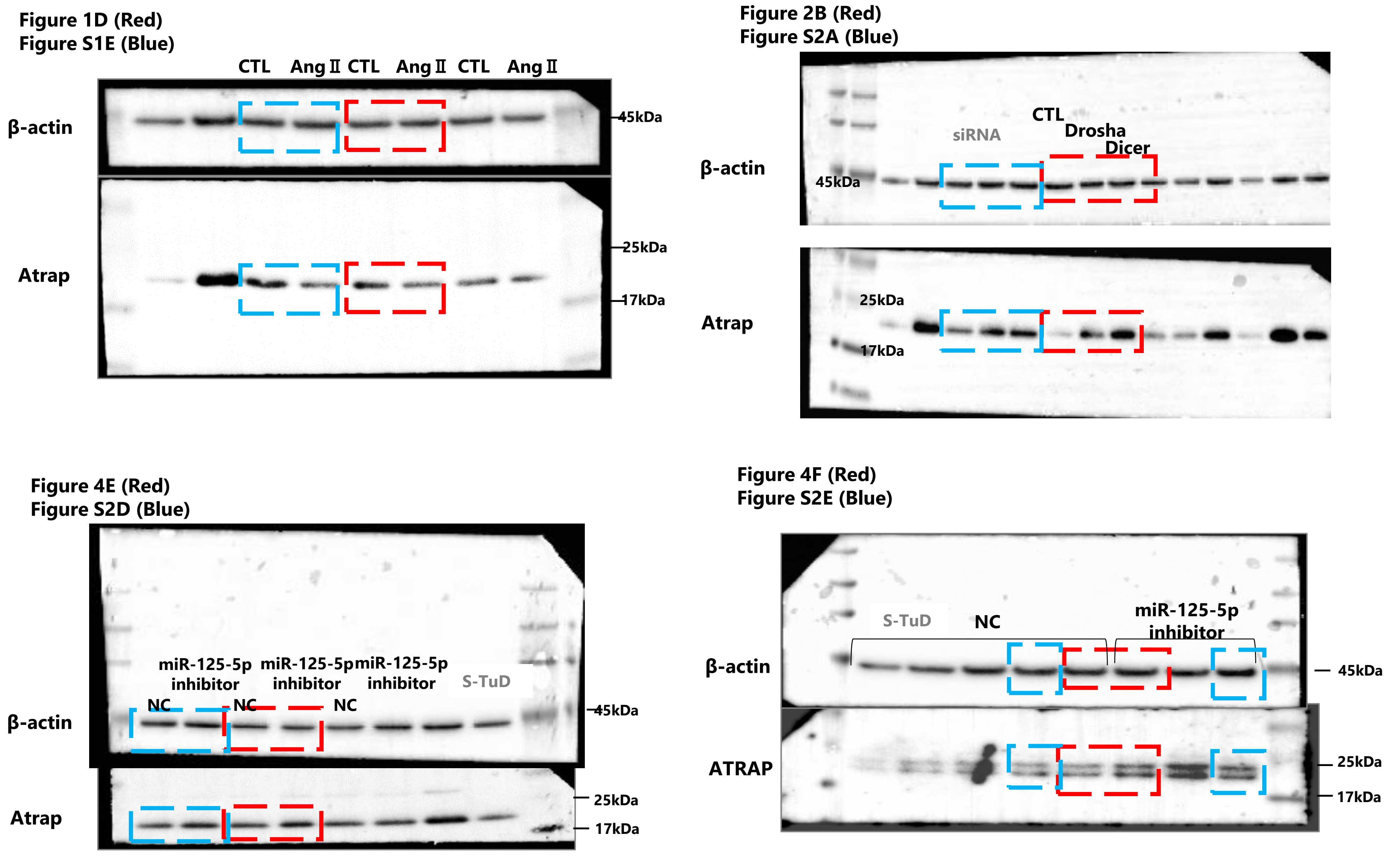

Supporting Figure 6

Figure 6A (Red)
Figure S3B (Blue)

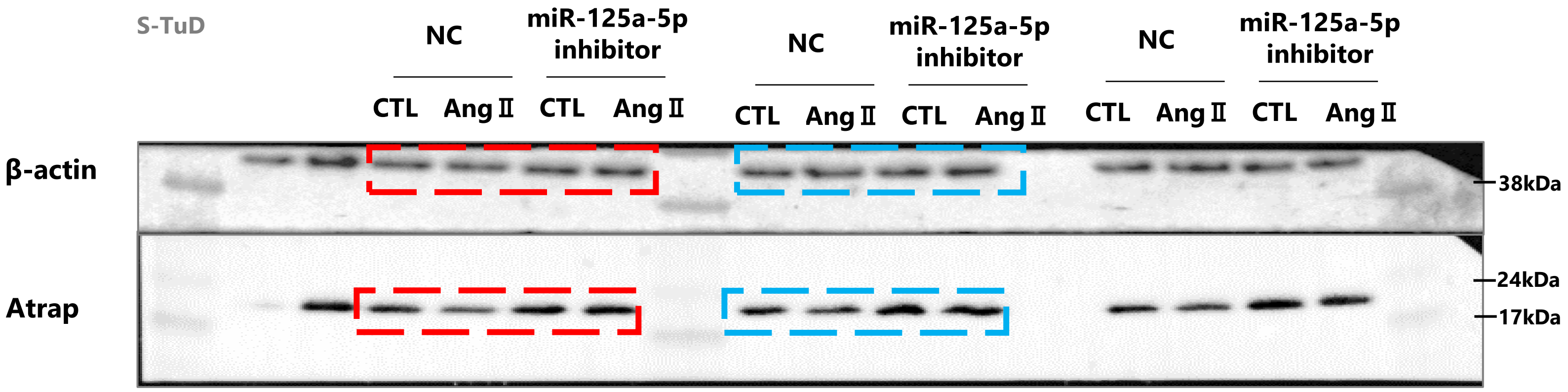

Figure 6C (Red)
Figure S3D (Blue)

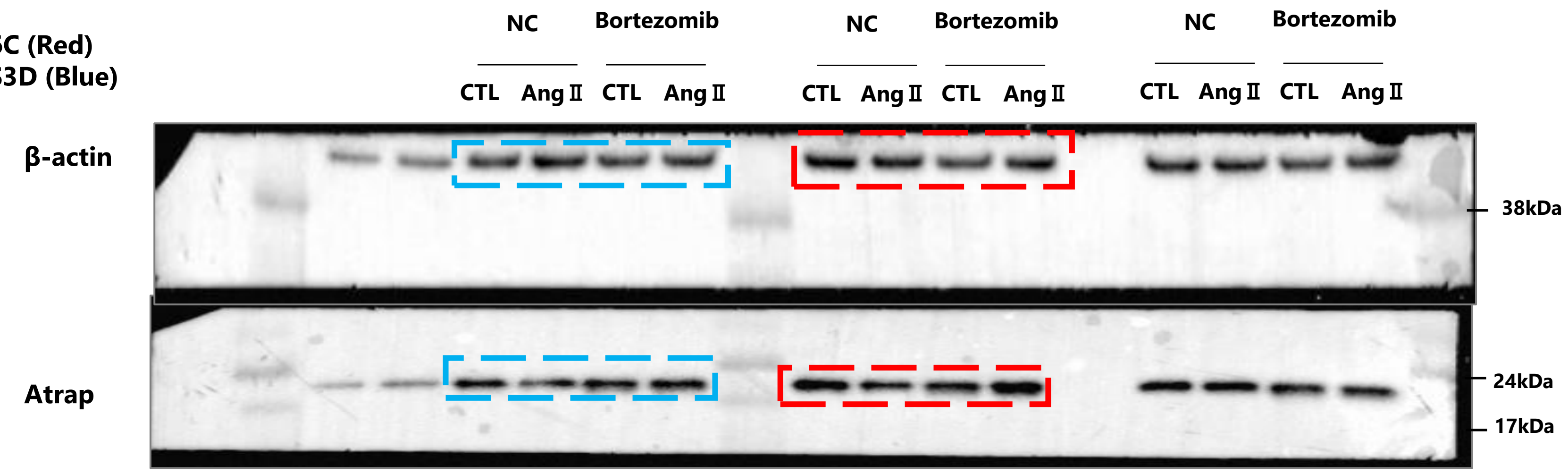

Supporting Figure 7

Figure 7B (Red)
Figure S4B (Red)
Figure S4C (Blue)

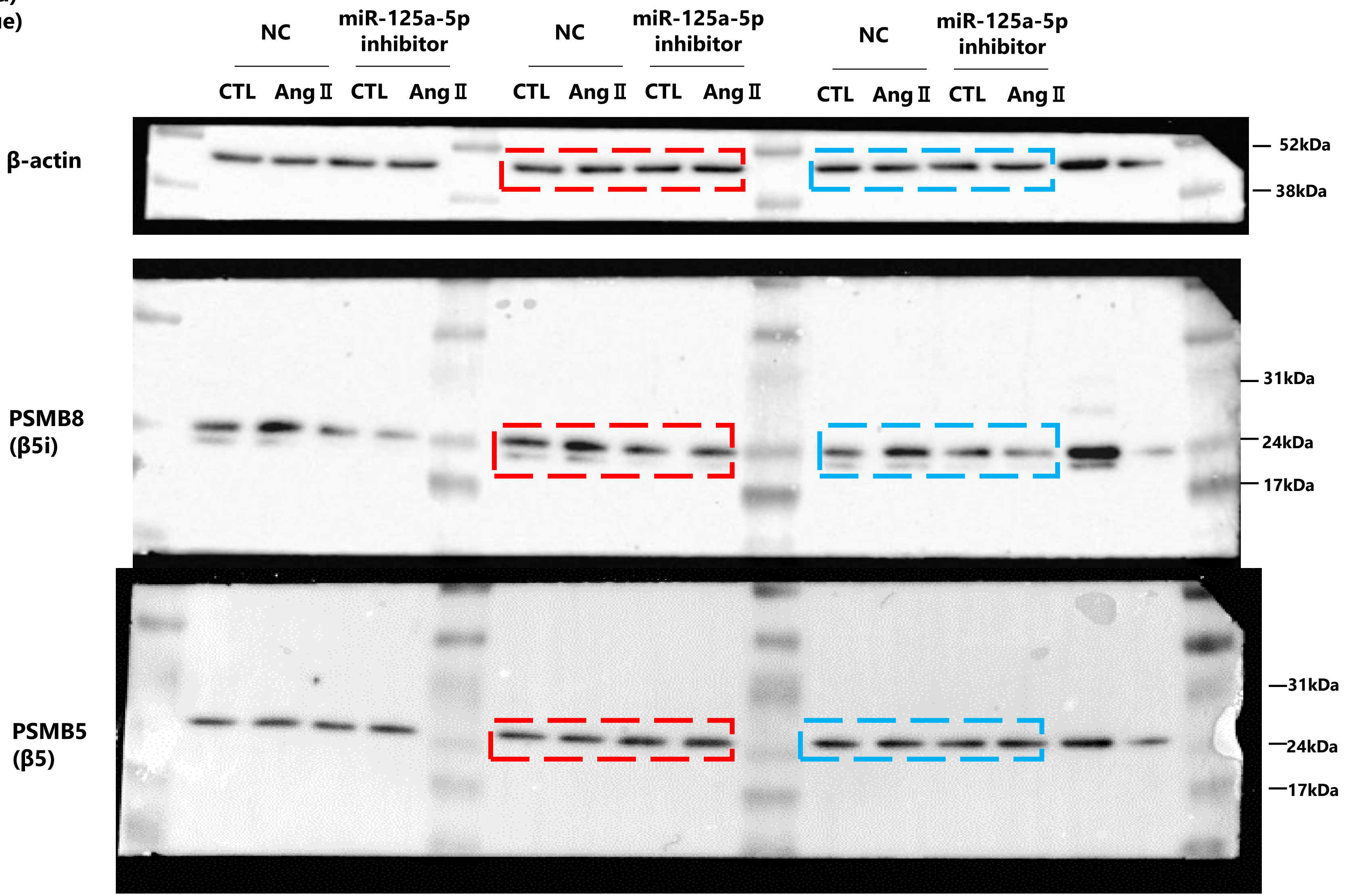

Supporting Figure 8

Figure 8C (Red)
Figure S4F (Blue)

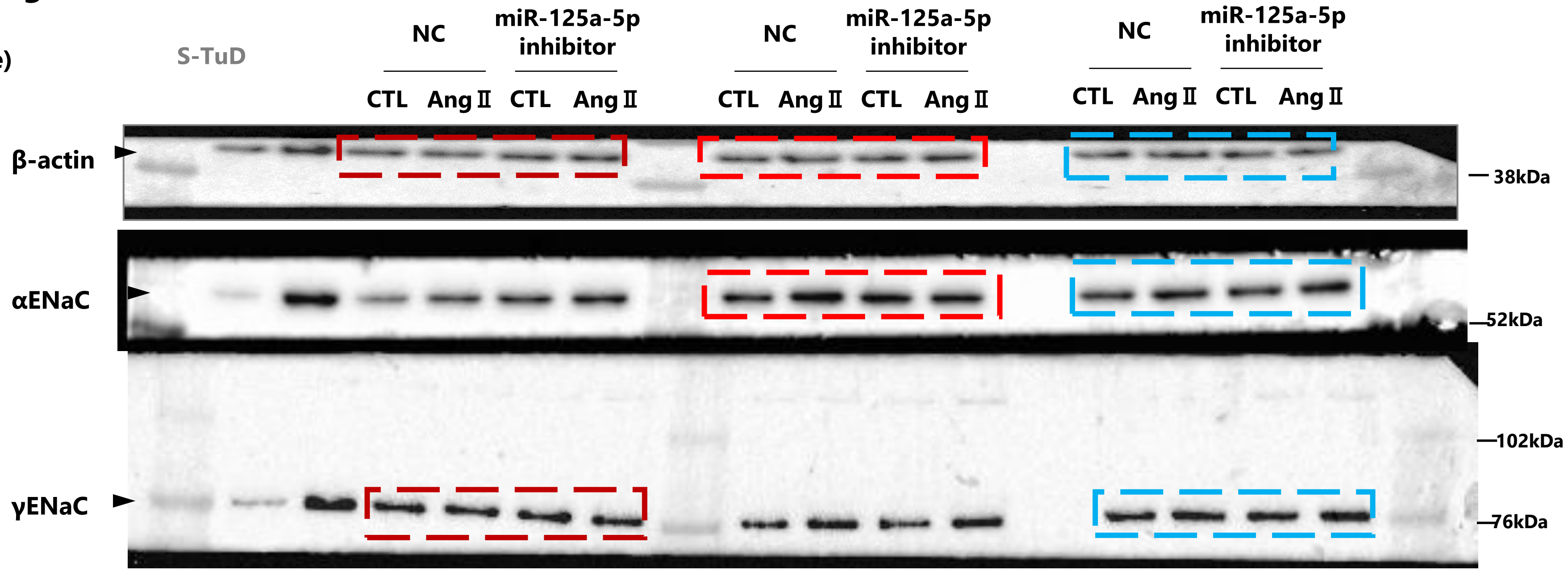

Figure 8D (Red)
Figure S4G (Blue)

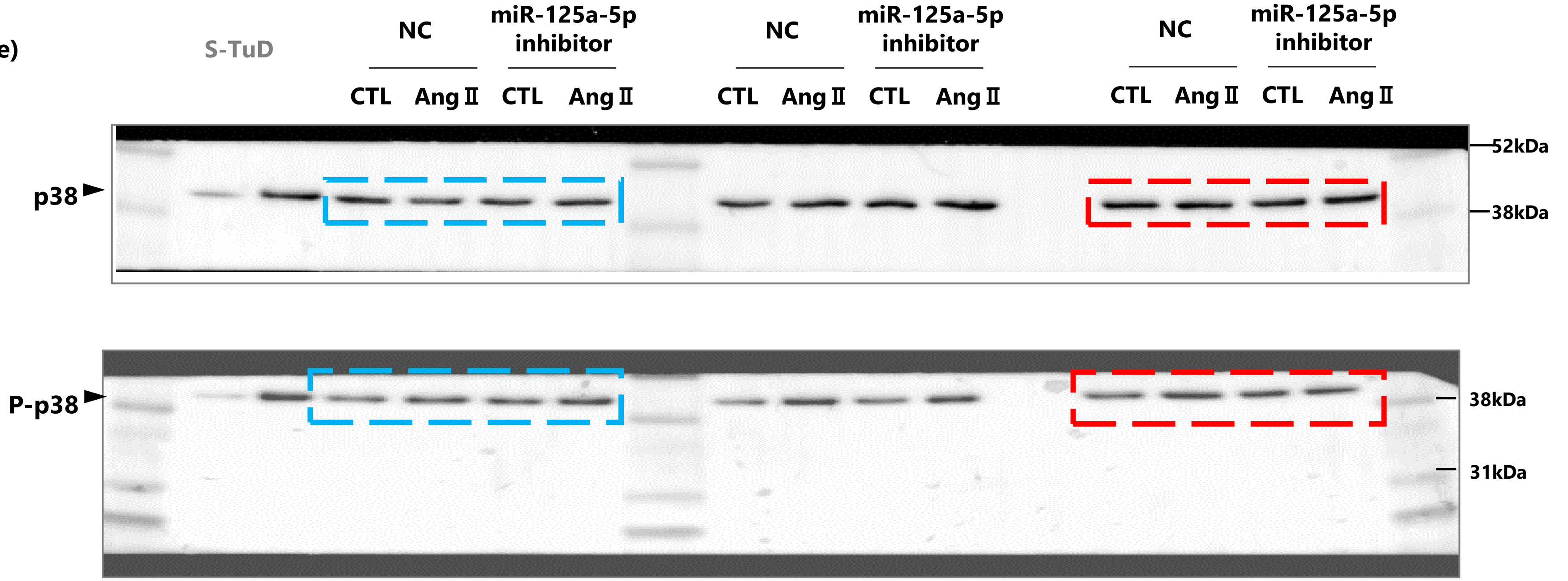

Supporting Figure 9

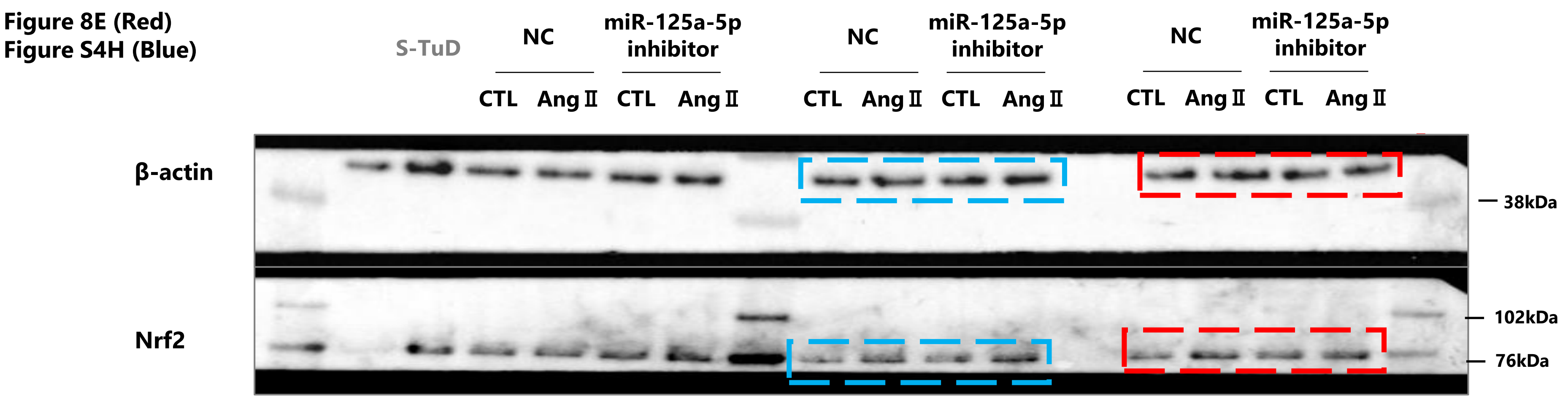

Supplement: Supporting Figures S1–S9 [file mmc1.pdf]
